# Anomalies of the aortic arch in dogs: evaluation with the use of multidetector computed tomography angiography and proposal of an extended classification scheme

**DOI:** 10.1186/s12917-021-03101-7

**Published:** 2021-12-16

**Authors:** Christiane Schorn, Nicolai Hildebrandt, Matthias Schneider, Sebastian Schaub

**Affiliations:** Justus-Liebig University Clinic for Small Animals, Frankfurter Strasse 114, 35390 Giessen, Germany

**Keywords:** Vascular ring anomaly, Persistent right aortic arch, Persistent ductus arteriosus, Vascular anomaly

## Abstract

**Background:**

Congenital anomalies of the aortic arch are important as they may be associated with vascular ring anomalies. The most common vascular ring anomaly in dogs is a persistent right aortic arch. However, published data of the distribution of the different types of vascular ring anomalies and other aortic arch anomalies are lacking. The objective of this retrospective descriptive study was to evaluate both the prevalence and the different types of aortic arch anomalies that can be detected using thoracic computed tomography (CT) examination. Archived thoracic CT examinations acquired between 2008 and 2020 at a single institution were retrospectively evaluated by 2 evaluators for the prevalence and type of aortic arch anomaly. Breed, age, and presenting complaint were obtained from the medical record system.

**Results:**

A total of 213 CT studies were evaluated; 21 dogs (21/213, 9.9%) showed a right aortic arch and a left ligamentum arteriosum with compression of the esophagus. The following incidental additional findings were detected: aberrant left subclavian artery (17/21, 76.2%), branching from the persistent ductus arteriosus (PDA) (1/21, 4.8%), left-sided brachiocephalic trunk (3/21, 14.3%), bicarotid trunk (17/21, 81.0%), double aortic arch (1/21, 4.8%). One hundred ninety two dogs (192/213, 90.1%) showed a left aortic arch without esophageal compression. The following additional abnormalities were obtained in those dogs with left aortic arch: aberrant right subclavian artery (3/192, 1.6%) without clinical signs of esophageal compression, aberrant vessel branching from the aorta into the left caudal lung lobe (2/192, 1.0%), focal dilatation of the left or right subclavian artery (2/192, 1.0%), bicarotid trunk (1/192, 0.5%).

**Conclusion:**

Similar to previous studies an aberrant left subclavian artery is the most common additional finding in dogs with persistent right aortic arch. Newly, a left-sided brachiocephalic trunk was identified in 14.3% of the dogs with a persistent right aortic arch; no additional compression was caused by the left sided brachiocephalic trunk. Similarly, aberrant right subclavian artery can be an incidental CT finding without causing compression of the esophagus.

## Background

**Fig. 1:**
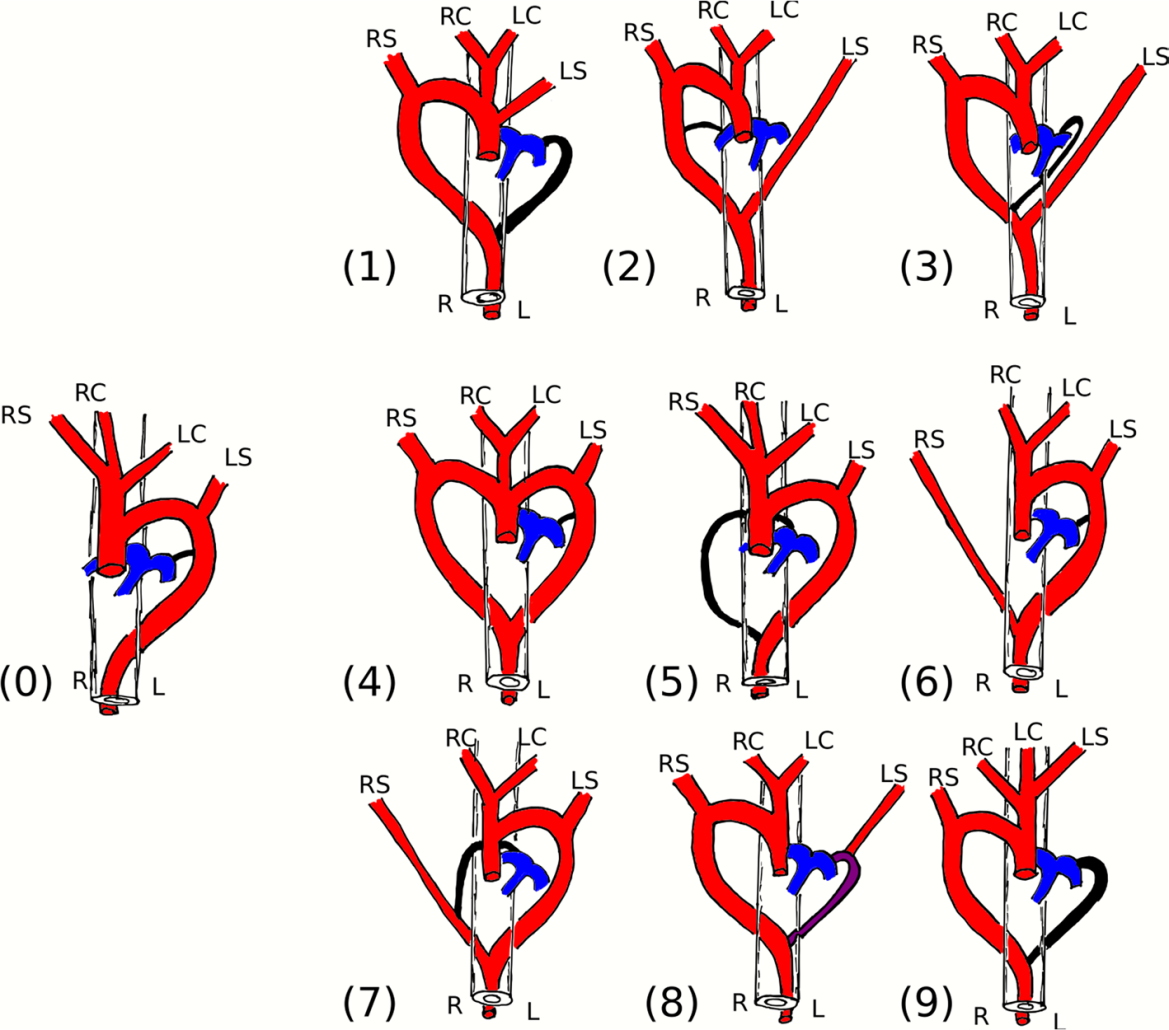
Classification scheme for aortic arch anomalies leading to a vascular ring. All types can potentially cause esophageal compression. Esophageal compression is less likely in Type 2 and 6. Legend: (0) normal anatomy of the aortic arch; (1) Type 1, persistent right aortic arch with left ligamentum arteriosum; (2) Type 2, persistent right aortic arch with  persistent left subclavian artery; (3) Type 3, persistent right aortic arch with left ligamentum arteriosum and left subclavian artery; (4) Type 4, double aortic arch; (5) Type 5, normal left aortic arch with right ligamentum arteriosum; (6) Type 6 normal left aortic arch with persistent right subclavian artery; (7) Type 7, normal left aortic arch with persistent right ligamentum arteriosum and right subclavian artery; (8) Type 8, right aortic arch with left subclavian branching from the PDA; (9) Type 9, right sided aortic arch with left-sided brachiocephalic trunk including both carotid arteries and the left subclavian artery referred to as “mirror” image. The blue region represents the pulmonary arteries and the black region the ligamentum arteriosum. Type 1 to 7 previously described by Ellison G. Vascular Ring Anomalies in the dog and cat. Compendium on Continuing Education for the Practicing Veterinarian -North American Edition-. 1980;2:693–705

Anomalies of the aortic arch are important to recognize because they may be associated with vascular ring anomalies [1]. A vascular ring anomaly is defined as a congenital disorder of the aortic vasculature that involves complete or partial encircling of the esophagus and trachea with secondary esophageal compression [1–3]. Due to esophageal compression, affected dogs show postprandial regurgitation of solid food [3]. Contrast-enhanced computed tomography allows an accurate anatomic diagnosis of vascular anomalies of the aortic arch [4–6]. Persistent right aortic arch is one of the most common ring anomalies in dogs, with a prevalence of about 7% [7, 8]. Pure-breed dogs seem to be more often affected than mixed-breed dogs, with German Shepherd dogs being overrepresented [7, 8]. Embryologically, the aorta develops out of an aortic sac, which is connected to the bilateral dorsal aortae by six paired aortic arches that develop bilaterally to the pharynx [9]. The dorsal aortae form a ring. During embryogenesis, the first, second, and fifth aortic arch degenerate. The third arches bilaterally become the common carotid arteries, and the dorsal aortae between the third and fourth arch degenerate [9, 10]. The left fourth aortic arch forms the ascending aorta, and the right fourth aortic arch contributes, together with the seventh intersegmental artery, to the right subclavian artery [9, 10]. The brachiocephalic trunk originates from the aortic sac and the third and fourth aortic arch. The sixth aortic arches become the left and right pulmonary arteries and continue as left and right ductus arteriosus. The right ductus arteriosus disappears prenatally, the left ductus arteriosus closes postnatally, and the ligamentum arteriosum remains [10]. The right dorsal aorta degenerates caudally to the right subclavian artery, and the left dorsal aorta forms the descending aorta [9]. When the right fourth aortic arch and the right dorsal aorta enlarge instead of the left, persistent right aortic arch develops [10]. When the right fourth aortic arch and the right dorsal aorta enlarge instead of the left, persistent right aortic arch develops [10]. Physiologically, the right ductus arteriosus degenerates, and the left ductus arteriosus persists, forming a connection between the left pulmonary artery and the abnormal right aortic arch, leading to constriction of the esophagus [10]. This is the most common type of vascular ring anomaly in dogs, classified as Type 1 [9]. In current literature, seven different types of vascular ring anomalies - causing variable degree of esophageal compression - have been classified in dogs: Type 1, persistent right aortic arch with persistent left ligamentum arteriosum; Type 2, persistent right aortic arch with persistent left subclavian artery; Type 3, persistent right aortic arch with persistent left ligamentum arteriosum and left subclavian artery; Type 4, double aortic arch; Type 5, normal left aortic arch with persistent right ligamentum arteriosum; Type 6, normal left aortic arch with persistent right subclavian artery; Type 7, normal left aortic arch with persistent right ligamentum arteriosum and right subclavian artery [9, 10] (Fig. 1). Because of the increased availability of computed tomography (CT), more thoracic CT studies are available, and aortic arch anomalies can be detected even in cases without clinical signs of vascular ring anomaly. To the authors knowledge, there is no data describing the frequency of aortic arch anomalies that could be detected during standard computed tomography of the thoracic cavity at one single institution. Therefore, the purpose of the study was to retrospectively evaluate thoracic CT studies and to describe the types and frequency of aortic arch anomalies identified in a group of dogs that underwent thoracic CT for suspicion of vascular ring anomaly and reasons unrelated to vascular ring anomalies.

## Material and methods

The study used a retrospective, case series descriptive design. All thoracic CT examinations of dogs present in the data base of the Justus Liebig University Clinic for small animals dating from January 2008 to December 2020 were reevaluated. Only CT examinations, which include pre- and post-intravenous iodinated contrast agent injection images were included. As a retrospective study, all data sets were acquired from clinical patients using standard veterinary practice, and no animal care and use protocol was required.

For dogs meeting the inclusion criteria, the following medical record data were documented: breed, sex, age at time of imaging, date of imaging, and reason for presentation.

### CT examination

The computed tomographic examinations were performed under general anesthesia. General anesthesia was induced with propofol and maintained with isoflurane by use of mechanical ventilation. All CT examinations were carried out using a 16-detector-row CT system (either SOMATOM Emotion, Siemens Healthcare, Erlangen Germany, or Diamond Select Brilliance, Philips Health Systems, Best, Netherlands). The CT scans were performed in sternal recumbency using a breath-hold technique was performed in 201 out of 213 dogs. For the breath-hold technique apnea was induced by manual hyperventilation and a positive pressure of 10 to 20 mm/Hg was maintained. Contrast medium (AccupaqueTM 300; GE Healthcare Buchler GmbH & Co. KG; Braunschweig; Germany; 300 mg J/ml) was injected intravenously via peripheral venous catheter at a dose of 2 ml/kg bodyweight, using a power injector (Medtron AG, Saarbrücken, Germany) at a maximum flow rate of 5 ml/sec, followed by a 5–10 ml saline flush at the same injection rate. In all cases, scans were delayed for 60 to 90 s after contrast medium administration in order to achieve a “late venous phase”. In 53 cases, arterial phase images were acquired using bolus tracking. Scanner setting was as follows: 1.5 mm slice thickness, a pitch of 0.8, tube rotation time 0.6 s, 130 kV, and 160 to 200 mA.

### Image evaluation

All data sets were reviewed by a first-year resident (C.S.) of the European College of Veterinary Diagnostic Imaging (ECVDI) and one ECVDI board-certified radiologist (S.S.), using the DICOM-viewing software (Horos v. 3.3.6, Los Angeles, California). Image orientation, window width and level could be adjusted by the reviewers according to personal preference. Additionally, the reviewers had the option to perform three-dimensional (3D) reconstructions of the datasets.

Only abnormalities affecting the aortic arch and the brachiocephalic trunk were documented. Each case was evaluated for pathologies concerning the aortic arch, including the right aortic arch, the existence of a right or left aberrant subclavian artery, patent ductus arteriosus (PDA), normal anatomy of the brachiocephalic trunk, and other findings. If a right aortic arch was present, dogs were whenever possible categorized into the previously described types [9, 10]. If none of the previously described types matched the CT findings origin and branching of any aberrant vessels were recorded. The diameter of the aberrant vessel was subjectively assessed and any increase in diameter distal to its origin was classified as dilatation.

The study population was divided into two groups one with and the other one without clinical signs of esophageal compression.

## Results

A total of 213 dogs met the inclusion criteria and the CT scans were evaluated. In 53 cases arterial and late venous phase was available, in the other cases only late venous phase images were available. Of the 213 dogs, twenty-one showed clinical signs referring to a vascular ring anomaly, such as regurgitation after food intake. The remaining 192 dogs underwent thoracic CT due to other reasons not related to vascular ring anomaly, such as exercise intolerance in 2 cases, spontaneous pneumothorax, lung diseases, or for metastatic screening and had no clinical signs consistent with vascular ring anomaly. In the following the results are presented for dogs with and without clinical signs of vascular ring anomaly.

### Dogs with clinical signs consistent with vascular ring anomaly

A total of 21 dogs showed clinical signs of suspected vascular ring anomaly. There were 15 female and 6 male dogs of the following breeds: 9 Labrador Retrievers, 4 German Shepherds, 2 mixed-breed dogs, 1 French Bulldog, 1 Gos d’Atura Català, 1 Husky, 1 Jack Russel Terrier, 1 Border Collie, and 1 Australian Shepherd. The mean age of the dogs was 2 months (range 6 weeks to 4 months). All 21 dogs showed a right aortic arch on CT examination, with secondary esophageal compression caused by the left ligamentum arteriosum. Esophageal compression by the left ligamentum arteriosum was confirmed surgically in all cases.

**Fig. 2:**
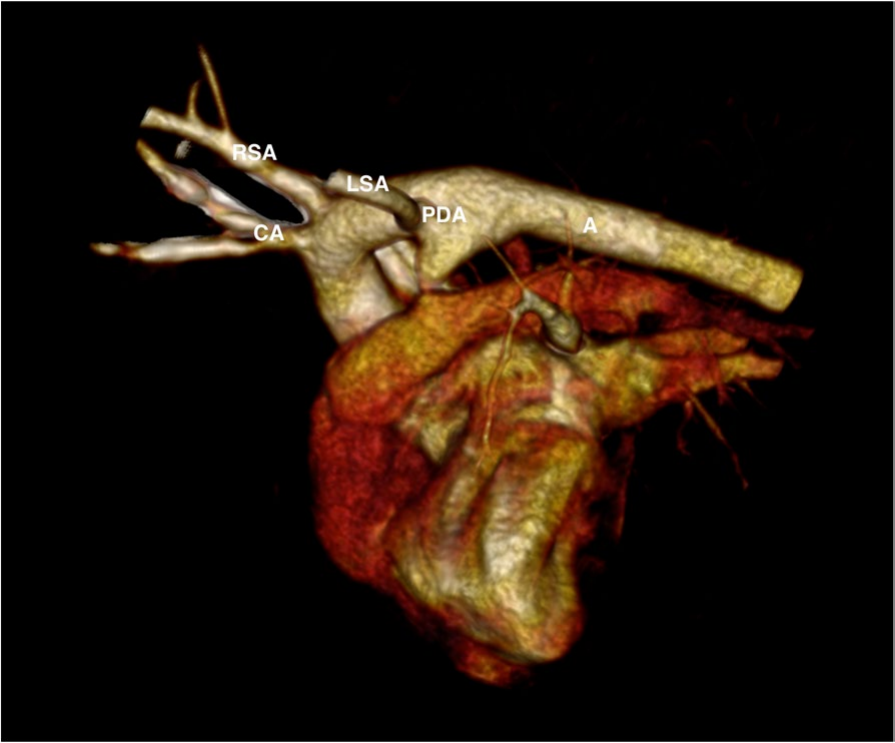
Volume rendered 3-dimensional CT reconstruction of an aberrant left subclavian artery (LSA) branching from the patent ductus arteriosus (PDA). Legend: A = Aorta, RSA = right subclavian artery, CA = carotid arteries

**Fig. 3:**
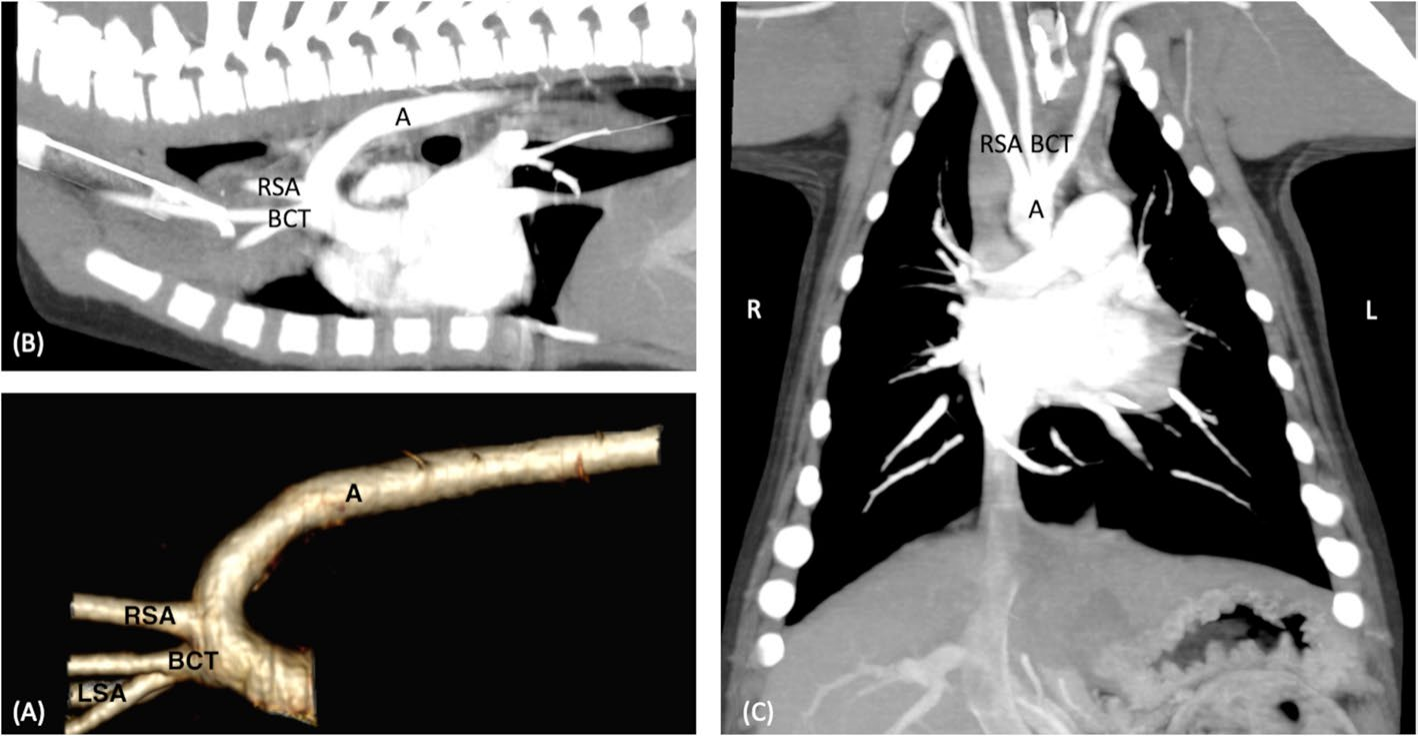
CT reconstruction of a left sided brachiocephalic trunk (BCT) composed of both carotid arteries (CA) and the left subclavian artery (LSA). The right subclavian artery (RSA) arose separately from the aorta. (**A**) Volume rendered 3-dimensional CT reconstruction, (B) sagittal plane MIP projection, (C) dorsal plane maximum intensity projection (MIP). Legend: A = Aorta

Concerning the previously published different types of vascular ring anomaly there were 16 dogs classified as Type 3 (persistent right aortic arch with persistent left ligamentum arteriosum and aberrant left subclavian artery). Of those 16 dogs 2 showed a branching of the aberrant left subclavian artery from the PDA (Fig. 2). In both cases PDA was closed surgically with dissection of the left subclavian artery. One dog was classified as Type 4 (double aortic arch). Three dogs did not fit on any of the previously described types. These 3 dogs showed a right aortic arch with a left-sided brachiocephalic trunk composed of both carotid arteries and the left subclavian artery. The right subclavian artery arose separately from the aorta (Fig. 3). Esophageal compression was caused by the persistent right aortic arch and the left ligamentum arteriosum in those three cases.

Beside those findings none of the 21 dogs with a right aortic arch showed a normal brachiocephalic trunk. The following additional abnormal branching patterns were found (Table 1).

**Table 1:** Number and percentages of arterial branching anomalies in dogs with right aortic arch. Esophageal compression present in all cases and was caused by the left sided ligamentum arteriosum

Anomalies in dogs with persistent right aortic arch (***n*** = 21)	Dogs	Percentage
Normal brachiocephalic trunk	0	0.0
Aberrant left subclavian artery (Type 3)	16	76.2
Bicarotid trunk	17	81.0
Left-sided brachiocephalic trunk including both carotid arteries and the left subclavian artery	3	14.3
Aberrant left subclavian artery branching from the PDA	2	9.5
Double aortic arch (Type 4)	1	4.8
Right subclavian artery had a separate branch form the right aortic arch	21	100
PDA visible	3	14.3

*PDA* Patent ductus arteriosus

### Dogs without clinical signs consistent with vascular ring anomaly

The remaining 192 cases did not show any clinical sign of vascular ring anomaly and underwent CT examination due to other reasons like exercise intolerance, pulmonary disease or for metastatic screening. The group consisted of 47 female, 42 female- spayed, 58 male and 45 male- neutered dogs. The mean age of the dogs was 6.4 years (range from 2 month to 16 years). One hundred fifty five were pure-breed dogs and 37 were mixed-breed dogs. Of those dogs 184 showed a normal aortic arch including a normal brachiocephalic trunk, without any evidence of esophageal compression. In the remaining 8 dogs changes of the branching or of the vessel diameter of either the aorta or the brachiocephalic trunk was detected (Table 2). In six of those eight dogs findings were considered as incidental without clinical relevance. The following findings were made on those 6 dogs: aberrant right subclavian artery without esophageal compression, focal dilatation of either the left or right subclavian artery, bicarotid trunk arising from the brachiocephalic trunk.

**Table 2:** Number and percentages of aortic and brachiocephalic trunk anomalies found in dogs with a left aortic arch

Aortic arch and brachiocephalic trunk anomalies in dogs with left aortic arch (***n*** = 192)	Dogs	Percentage
Aberrant vessel from the aorta draining into the left caudal lung lobe	2	1.0
Aberrant right subclavian artery	3	1.6
Focal dilatation of the left subclavian artery	1	0.5
Focal dilatation of the right subclavian artery (Fig. 5)	1	0.5
Bicarotid trunk arising from the brachiocephalic trunk	1	0.5

**Fig. 4:**
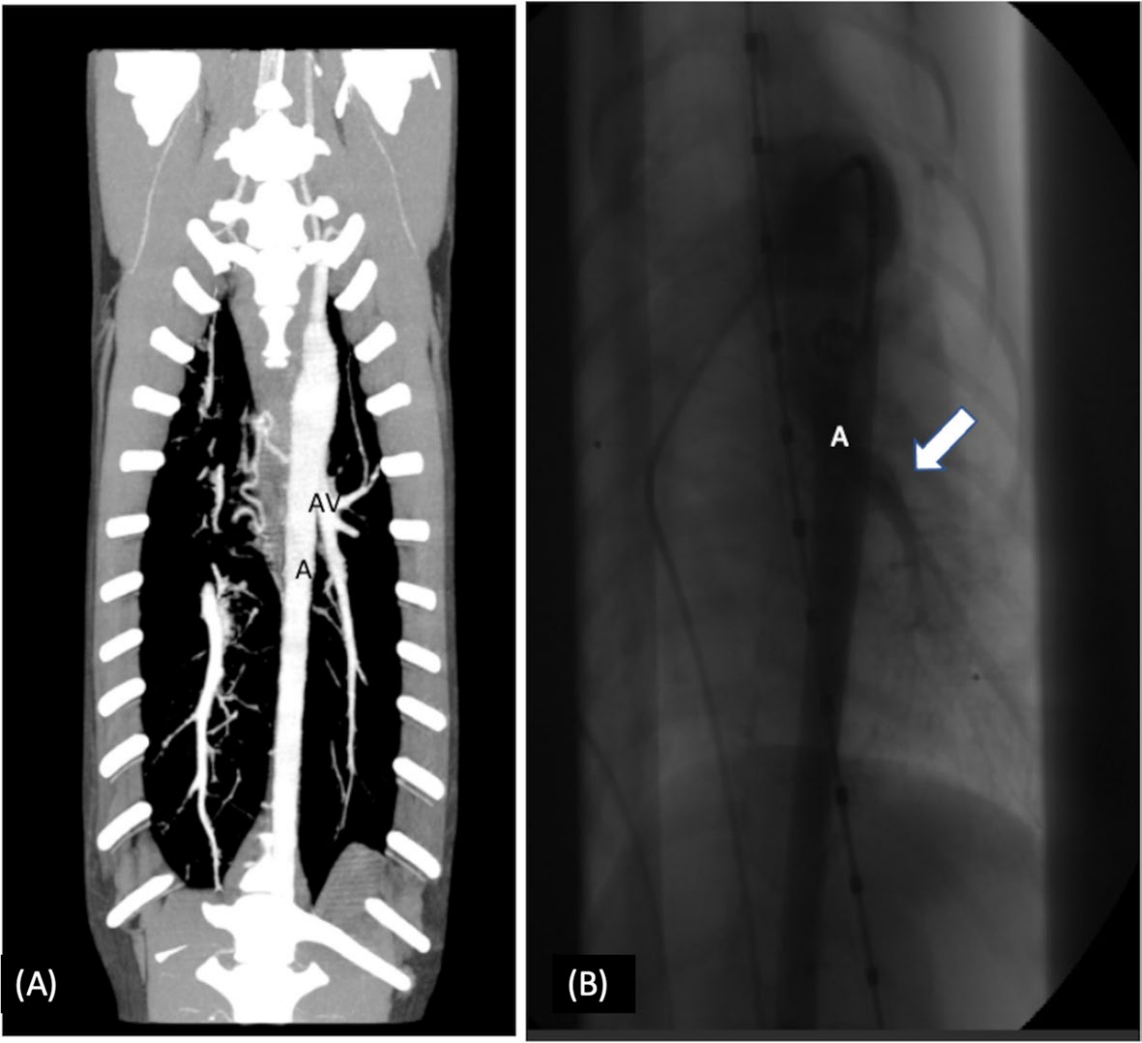
CT reconstruction of an aberrant arterial vessel (AV), arising from the left sided aorta (A) draining into the left caudal lung lobe, (A) dorsal plane MIP projection, (C) Angiography, arrow showing the AV, contrast enhancement occurs at the time of injection into the aorta

The remaining two dogs were presented due to exercise intolerance. Dog’s signalment was one male 2-month-old Flat-Coated Retriever and one male 6-month-old Shetland Sheepdog. The CT examination revealed in both cases an aberrant, additional vessel arising from the aorta at the level of the fourth thoracic vertebral body (Fig. 4). The aberrant vessel was running caudally and ventrally, draining into the left caudal lung lobe with subsequent systemic to pulmonary connection. Both dogs showed a persistent ductus arteriosus. The Flat-Coated Retriever presented a hypoplastic pulmonary artery on the left side as well as a moderately reduced lung volume on the left side compared subjectively to the right side. Nevertheless, both pulmonary arteries were detectable in both cases. The aberrant vessel in the 6-month-old Shetland Sheepdog as well as the PDA was closed via catheter embolization. The second dog was lost to follow-up.

**Fig. 5:**
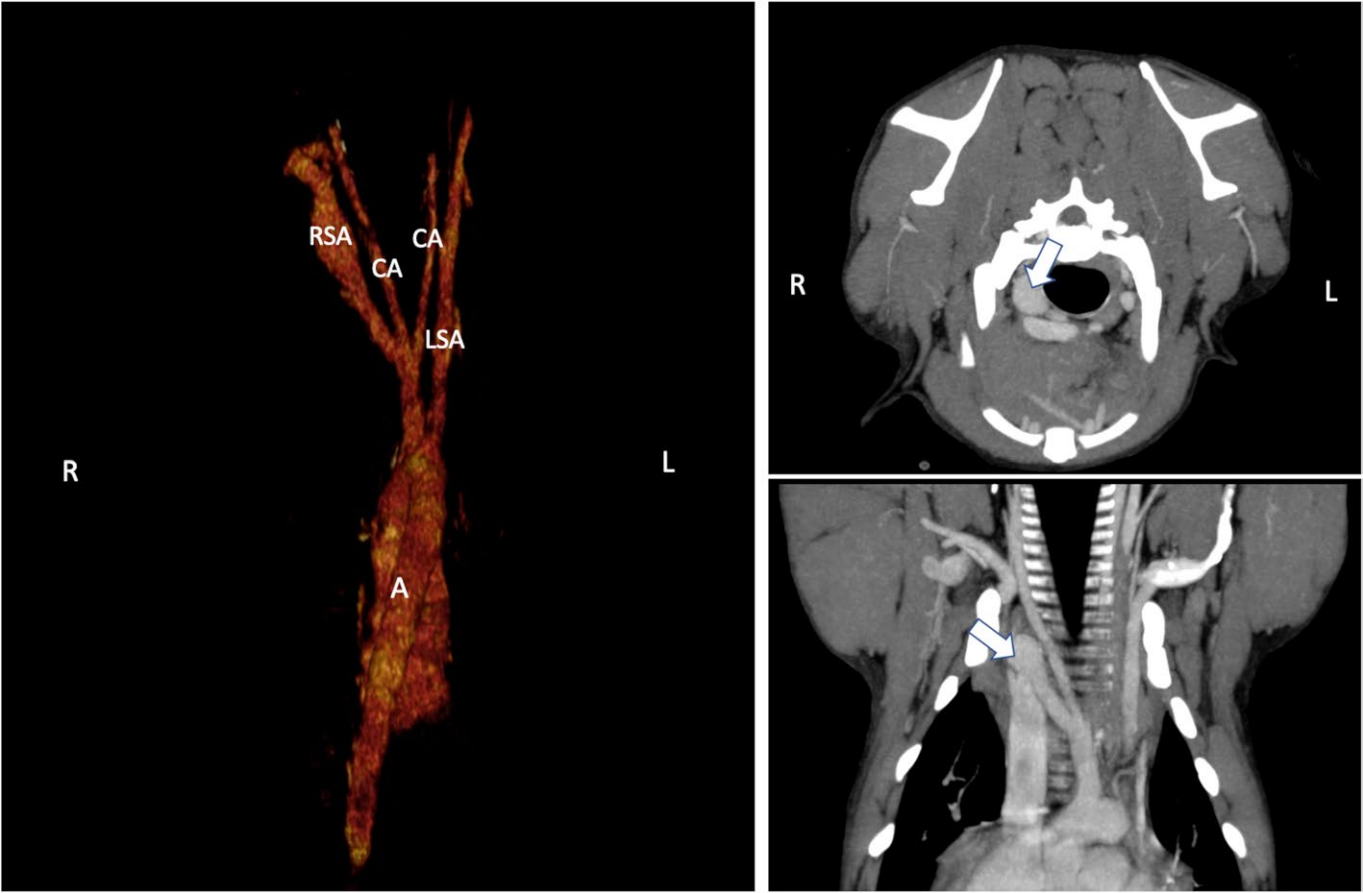
**A** Volume rendered 3-dimensional CT reconstruction of showed focal moderate dilation of the right subclavian artery (**B**) transverse plane and (**C**) dorsal plane at the level of the focal dilatation of the right subclavian, arrow showing dilatation. Legend: A = Aorta, RSA = right subclavian artery, LSA = left subclavian artery, CA = carotid artery

## Discussion

To the authors knowledge, this is the first paper both systematically describing variations of the aortic arch that can be found during thoracic CT examination in a group of dogs and comparing them to previously described types. In the study population, 9.9% showed a persistent right aortic arch; these findings are consistent with previously published data [7, 8]. In contrast to other studies, German Shepherd dogs were not overrepresented in the study population [6, 7]. The most affected breed in the current paper was the Labrador Retriever dog, representing 42.9% of the cases. Herewith the Labrador Retriever dog seems to be overrepresented at least in our hospital population. In addition to the initially described 7 different types of vascular ring anomalies, [9] a previously published review mentioned two further types [3]. The first additional type mentioned in the review has been characterized in a single surgical case report in 1979. The authors described an aberrant branch of the aorta arising 3 cm caudal to the left subclavian artery, passing anteromedially and draining into the brachiocephalic artery [11]. Considering the long course of the described vessel, a complete vascular ring appears less likely, and the described vessel represents most likely an aberrant right subclavian artery. The second additional mentioned type of vascular ring anomaly has also been found surgically and characterized as an incomplete esophageal constriction due to the first right intercostal artery branching directly dorsal from the aorta instead of branching from the costocervical trunk [12]. Considering the incomplete vascular ring in both cases, the two proposed additional types represent more likely aberrant aortic vessels rather than classical vascular ring anomalies. Therefore, they were not included into the classification scheme proposed in the current article. Persistent right aortic arch with persistent left ligamentum arteriosum and left subclavian artery (Type 3) was the most frequently detected ring anomaly, affecting 76.2% of all dogs with right aortic arch. Type 1 anomaly, without additional vascular changes, was not detected in any dog. This contrasts with previously published data describing Type 1 as the most common type [9]. Type 1 is embryologically less likely to occur since right aortic arch results from regression of the left fourth arch between the left carotid artery and the left subclavian artery, resulting in the left subclavian artery being the last one branching from the aorta [1]. In a more recently published study, an aberrant left subclavian artery was detected surgically or postmortem in 33% of the cases [13]. Another study describing CT features of dogs with right aortic arch revealed an aberrant left subclavian artery in 60% of the cases [4]. The lower percentages of aberrant left subclavian artery, published in older literature, are probably due to the fact that the final diagnosis was exclusively made by surgery. In some dogs, retroesophageal left subclavian artery as well as other additional vascular anomalies are not obvious and may not have been identified by the surgeon. Therefore, CT examination appears to be more accurate in detecting an aberrant left subclavian artery than surgery alone as surgery may underestimate additional vascular abnormalities. The clinical relevance of an aberrant left subclavian artery is unclear and may not contribute to esophageal compression, probably because of its more dorsally and near-midline origin [6]. Nevertheless, surgeons should be aware of the presence of aberrant left subclavian artery to assess for possible constriction during thoracotomy.

Normal left aortic arch with persistent right subclavian artery (Type 6 anomaly) was detected in three dogs without clinical signs suspicious for vascular ring anomaly and occurred as an incidental finding in the CT examination of the thoracic cavity performed due to other reasons. Aberrant right subclavian artery is usually associated with normal left aortic arch [10]. Embryologically aberrant right subclavian artery occurs when the right dorsal aorta, cranial to the subclavian artery, abnormally degenerates. Consequently, the distal right aorta, instead of the right fourth arch, becomes the base of the right subclavian artery, which is linked caudally to the left aorta. Therefore, the right aberrant subclavian artery is the last branch leaving the aortic arch [1, 14]. In human medicine, left aortic arch with aberrant right subclavian artery is the most common congenital malformation of the aortic arch, with a prevalence described between 0.5 and 2%, leading to esophageal compression in about 10% of these cases [1, 15]. In veterinary medicine, only few case reports exist, and there are no data describing the overall prevalence [14, 16–19]. The clinical relevance of aberrant right subclavian artery in dogs is unclear, with some case reports describing clinical syndromes such as dysphagia and regurgitation [11, 13–16]. The data collected in the present study shows that an aberrant right subclavian artery in dogs, as it is reported in human medicine, can be an incidental finding without any clinical relevance. The prevalence of an aberrant right subclavian artery is 1.4% in the study population. Another incidental finding was a mild focal dilatation of the right and left subclavian artery, detected in two dogs without any clinical relevance. In human medicine, dilatation of the subclavian artery is defined as Kommerell’s diverticulum and characterized by focal dilatation near the origin from the aorta [4, 20, 21]. The dilatation of the subclavian artery recognized in the own population was more distally and can therefore not be defined as Kommerell’s diverticulum but may be assessed as a normal anatomical variant.

One main finding of the study was the left-sided brachiocephalic trunk, found in three dogs with right aortic arch. The left-sided brachiocephalic trunk was composed of the left subclavian artery and both carotid arteries and thus represented a complete reflection of the brachiocephalic trunk on the left side. To our knowledge, this type has not been previously described in dogs. In human medicine, this type has been described as right aortic arch with mirror image branching and is the second most common form of a right-sided aortic arch [1]. The findings of the current study show that a left-sided brachiocephalic trunk also occurs in dogs, with a prevalence of 14.3% of dogs with right aortic arch in the own study population. Therefore, complementing the current classification scheme by the new type is proposed (Fig. 1).

Two dogs with right aortic arch showed an aberrant left subclavian artery branching from the patent ductus arteriosus. This type of aortic malformation has been described in only two dogs before and, thus, appears to represent a rare variant [22, 23]. The malformation, which is defined by an isolated left subclavian artery, is caused by regression of the left arch at two segments cranially and caudally to the left subclavian artery [1]. Like the previously reported type, this variant has not yet been characterized and should be included in the new modulated classification scheme, which is proposed by the authors (Fig. 1). Regarding surgical treatment, in both cases, ligation and dissection of the left subclavian artery were performed. In human medicine occlusion or stenosis of the proximal subclavian artery results in reversal blood flow through the vertebral artery. Common clinical signs are vertigo, syncope, and intermittent claudication of the involved upper extremity; the syndrome is known as subclavian steel syndrome [24]. No clinical signs consistent with the described subclavian steel syndrome were detected in one of the two dogs following surgery. Nevertheless, surgeons should be aware of potential side effects, and dissection of the subclavian artery with subsequent anastomosis to the left carotid artery could be recommended to prevent subclavian steel syndrome. Beside the ductal origin of the subclavian artery, 3 more types of anomalous left subclavian artery anatomy are described previously and include isolation, hypoplasia and lateral origin from the aorta [23]. Concerning the own study population one dog showed a mild narrowing of the left aberrant left subclavian artery caudal to its origin from the aorta consistent with the findings previously described as left subclavian hypoplasia. In contrast to the previously described case no hypertrophy of the left intercostal arteries was detected.

Two dogs in the study showed an aberrant vessel branching from the left sided aortic arch, coursing caudally, and draining into the left caudal lung lobe. Systemic to pulmonary shunting vessels have been described in dogs in multiple case reports [25–30]. In most cases in the current veterinarian literature, systemic to pulmonary shunting is described as hypertrophy of the bronchoesophageal artery, with multiple tortuous shunt vessels [25–28]. Bronchoesophageal artery hyperplasia can be a congenital disorder or may be acquired due to long-standing hypoxic states or due to pulmonary artery flow reduction [28, 31]. The two patients in the current study showed no evidence of bronchoesophageal artery hyperplasia, and there was only a single linear shunt vessel visible, branching directly from the descending aorta. In the respective patients, both pulmonary arteries were physiologically detectable even though the left pulmonary artery in one dog was mildly hypoplastic. In contrast to other authors, no direct connection between the shunt vessel and the pulmonary artery was detected [32]. There was no evidence for acquired aortic to pulmonary shunt vessels in the current study population and, except for the PDA, no accompanying congenital cardiac anomalies were evident. In human medicine, persistence of the 5th aortic arch can lead to systemic to pulmonary connection by an aberrant vessel [1, 33, 34]. Physiologically, the 5th aortic arches are rudimentary vessels that quickly degenerate during embryogenesis and lie between the 4th and 6th aortic arch [1, 34]. As the herein described anomalous vessels originated from the descending aorta caudal to the PDA, representing a remnant of the 6th aortic arch, a persistent 5th aortic arch is considered unlikely in these cases. Therefore, the origin of the described vessels remains unclear and most likely represents an aberrant intercostal artery. Nevertheless, in human medicine an aberrant pulmonary artery branching from the aorta is described [32]. To the authors knowledge comparable cases in dogs have not yet been published yet. The herein described vessel most likely represent an aberrant nutritive pulmonary vessel or an additional aberrant pulmonary artery. Since occlusion of the vessel in one case did not lead to clinical signs, the clinical importance of the vessel is questionable.

Limitations of this study include the retrospective nature. Arterial phase-computed tomographic studies were not available in all cases because thoracic-computed tomography was often performed due to other reasons than suspected vascular abnormalities. Nevertheless, visualization of the aortic arch was good in the late-phase contrast study. The small group of dogs with right aortic arch did not allow generating epidemiological data in respect of the distribution of congenital aortic arch malformation in dogs. The own referring clinic has a large focus on cardiac patients, and therefore, patients with cardiac diseases may have been overrepresented in the current study population.

## Conclusions

This study describes aortic arch anomalies and their distribution in a population of dogs, detected during thoracic-computed tomography. The findings indicate that further classification of right aortic arch types is needed since two more - to date unclassified - types are present in dogs, leading to a total number of at least nine different types in dogs. Therefore, an extended classification scheme is proposed by the authors. Furthermore, the results indicate that the aberrant right subclavian artery can be an incidental finding in dogs with left aortic arch and does not cause clinical signs. Future studies are needed to determine whether the findings from this study remain valid even for a larger dog population.

## Data Availability

The datasets analyzed during the current study are available from the corresponding author upon reasonable request.

## Abbreviations

CT: Computed tomography
PDA: Persistent ductus arteriosus
